# Superstructures with cyclodextrins: chemistry and applications III

**DOI:** 10.3762/bjoc.12.91

**Published:** 2016-05-10

**Authors:** Gerhard Wenz, Eric Monflier

**Affiliations:** 1 Saarland University, Organic Macromolecular Chemistry, Campus C4 2, 66123 Saarbrücken, Germany; 2 Université d’Artois, Unité de Catalyse et de Chimie du Solide (UCCS), CNRS, UMR 8181, Rue Jean Souvraz, SP 18, 62307 Lens, France

**Keywords:** cyclodextrins

Cyclodextrins (CDs) are of increasing scientific and commercial interest because they are readily available, harmless and well-defined, forming sophisticated supramolecular structures in aqueous media. More than 2,300 publications and more than 1,200 patents appeared in 2015 in which CDs played a significant role. As exemplified in the following, CD chemistry has developed into a very attractive field of research.

The cell-penetrating peptide octa-arginine was conjugated to methylated β-CD. The resulting biofunctionalized host was able to transport a porphyrin sulfonate into HeLa cells used in photodynamic therapy [1]. Furthermore, it was found that lactose-appended β-CD lowers the cholesterol level in HepG2 cells – a result that is relevant for the treatment of Niemann–Pick-type C disease [2]. Metal-organic frameworks (MOFs) created from native CDs can be applied for the separation of isomeric xylenes or styrene from ethylbenzene [3–4]. Highly porous networks were synthesized by crosslinking β-CD with tetrafluoroterephthalonitrile. These networks were able to almost completely remove pollutants (e.g., bisphenol A) within a short time from wastewater [5]. Stimuli-responsive nanoparticles were assembled by the group of B. J. Ravoo using host–guest interactions between polyethyleneimine grafted with β-CD and a polyamidoamine dendrimer decorated with ferrocene. The formation of the superstructures was reversible by electrochemical oxidation of the ferrocene moieties [6].

Furthermore, significant progress has been achieved in the synthesis of CD polyrotaxanes. The one-pot and one-step polyrotaxane synthesis of β-CDs was performed in aqueous solution by employing a click reaction [7] as well as by radical copolymerization [8]. Slide-ring gels, synthesized by the group of K. Ito through the crosslinking of the hydroxy groups of CD polyrotaxanes, showed peculiar material properties [9] such as extreme stretchability [10]. These mobile networks are potentially useful as self-healing coatings [11].

Finally, recent results have shown that CDs are precious molecular tools that can be used for the design of highly active heterogeneous catalysts. Accordingly, the catalytic transformation of bio-oil has been achieved over Cu/MCM-41 and Cu/Kit-6 catalysts obtained by a CD assisted co-impregnation method [12]. Furthermore, the combination of template-directed colloidal self-assembly with a CD-assisted impregnation enabled to prepare highly active and selective Ru/TiO_2_ catalysts for the hydrogenation of oleic acid methyl ester [13].

The latest results of CD chemistry have been exchanged at two international conferences in 2015. The *Joint Conference of 8^th^ Asian Cyclodextrin Conference and 32^nd^ Cyclodextrin Symposium* with 200 participants, 34 oral presentations and over 100 poster contributions took place in Kumamoto, Japan on May 14–16, 2015. The *4^th^ European Conference on Cyclodextrins* was held in Lille, France on October 6–9, 2015 and brought together 170 researchers from 29 countries across the world. This congress with 130 presentations covered all fields of cyclodextrin research such as pharmacy, materials, catalysis, remediation, formulation and chemical analysis.

Some remarkable original papers and reviews are published in this Thematic Series of the *Beilstein Journal of Organic Chemistry*. In particular, the group of F. Estour has published an update on the current application of CDs for the detoxification of organophosphorus compounds [14]. In fact, the bowl-shaped structure of cyclodextrins is ideally suited for the design of new chemical scavengers for organophosphorus compounds, allowing these highly dangerous substances to be trapped and hydrolyzed before they reach their biological target. G. Cravotto et al. have reported on the recent advances in enabling technologies and green processes in CD chemistry [15]. By increasing heat and mass transfer, the protocols (assisted by microwaves, ultrasound or ball mills) appeared to be energetically more efficient as compared to classical synthetic tools.

Gerhard Wenz and Eric Monflier

Saarbrücken, Lens, April 2016
